# The common redstart as a suitable model to study cuckoo-host coevolution in a unique ecological context

**DOI:** 10.1186/s12862-016-0835-5

**Published:** 2016-11-25

**Authors:** Peter Samaš, Jarkko Rutila, Tomáš Grim

**Affiliations:** 1 Department of Zoology and Laboratory of Ornithology, Palacky University, 17. listopadu 50, Olomouc, 77146 Czech Republic; 2 Kannelkatu 5 as 26, Lappeenranta, Finland

**Keywords:** Arms-races, Co-evolution, Defence, Mimicry

## Abstract

**Background:**

Co-evolutionary arms-races result in spatio-temporally dynamic relationships between interacting species, e.g., brood parasites and their avian hosts. However, majority of avian co-evolutionary studies are limited to “snap-shots” of a single breeding season in an open-nesting host. In a long-term study (11 breeding seasons), we explored a unique system between the brood parasitic common cuckoo (*Cuculus canorus*) and its host, the common redstart (*Phoenicurus phoenicurus*) which is exceptional among all cuckoo hosts due to being a cavity nester. Conditions in cavities are different from open nests, e.g., lower risks of predation, more favourable microclimate, increased risks of unsuccessful eviction of host offspring by the cuckoo nestling. Different conditions in cavities thus can be expected to shape parasite-host coevolution differently from what is typically studied in open nesting hosts.

**Results:**

In our highly parasitised nest-box population (32.5%, *n* = 569 nests) only 35.7% of cuckoo eggs were laid into the nest cup and incubated by redstarts. Host nests shifted availability to later into the breeding season from 2006 to 2016 and cuckoos followed this trend by also shifting their timing of parasitism. Although previous studies revealed that redstarts selectively eject experimental non-mimetic eggs (desertion was not a specific response to foreign eggs), the hosts never ejected naturally-laid cuckoo eggs or cuckoo eggs cross-fostered into naturally non-parasitised nests. We solve the long-standing debate about the origin of cuckoo eggs found on the nest rim: we gained the first direct video-recording evidence that eggs found on the nest rim were mislaid by parasites and not ejected by hosts. Naturally-parasitised nests were deserted more often (18.6%) than control non-parasitized nests (5.6%) or nests artificially parasitised by us (1.4%). This suggests that the sight of the laying cuckoo female is the primary cue that triggers egg rejection (by desertion) in this host. Review of data from this and other study sites (10 populations, *n* = 853 experiments) demonstrates high variability in rejection rates and shows that populations facing higher parasitism rates reject parasitic eggs with higher frequencies. Surprisingly, cuckoo chicks either growing solitarily or with redstart chicks did not differ in their fledging success.

**Conclusions:**

We suggest that the redstart is an ideal model system to study the flexibility and limits of brood parasite-host co-evolution in an extreme ecological setting.

**Electronic supplementary material:**

The online version of this article (doi:10.1186/s12862-016-0835-5) contains supplementary material, which is available to authorized users.

## Background

Brood parasitic birds and their hosts provide suitable model systems to study co-evolutionary interactions [1]. Parasites diminish their hosts breeding success and hosts defend against such detrimental effects which causes long-term oscillations of adaptations and counter-adaptations [2, 3]. The common cuckoo (*Cuculus canorus*) and its hosts have been thoroughly studied under this scenario [4]. However, the vast majority of cuckoo studies have been done in open nesting hosts [5–7], and mainly those breeding in reed beds [8–14]. Additionally, theoretical models, their assumptions and predictions have been explicitly based on studies of open nesting hosts [3, 15].

However, there is one peculiar cuckoo host, the redstart (*Phoenicurus phoenicurus*) which is a cavity nester [16]. The redstart is parasitised by a very distinct cuckoo genetic race that lays highly mimetic blue eggs [17, 18]. Based on museum egg-collections, distribution of this cuckoo race is currently limited to Fennoscandia (from south and central Finland to south-eastern Norway) [19]. Just a century ago, blue cuckoo eggs were often reported in redstart nests from Central Europe [20] but we are aware of no more recent reports. The redstart distribution is much wider, spanning all over Europe and reaching up to Siberia [19]. In contrast to all other regular cuckoo hosts, the cuckoo race parasitising redstarts shows low eviction success [16, 21] and cuckoo chicks cohabiting with redstart chicks consequently show low fitness [22]. These experimental studies suggest that cavity nesting importantly affects cuckoo-host interactions and might shape the arms-race to an alternate trajectory that differs from that in open-nesting hosts; thus, theoretical models might benefit from including specifics of this unique system to address the flexibility and limits of cuckoo biology. However, this research has thus far been neglected [23], partly because of the scarcity of data, especially under long-term natural non-manipulated conditions [24].

Here, we present detailed, long-term data from a redstart population in Finnish Karelia using methods and data presentations to make our study quantitatively directly comparable with previous detailed studies [16, 24, 25]. Nest box sizes, their positions on trees, and distances between them were roughly similar in our and these studies, making our conclusions reasonably comparable (cf. information below with [16, 24, 25]). Due to its geographical position, our study site provides a well-isolated meta-replicate (sensu [26]; see Methods) that can be used to assess the generality of findings of previous studies. Such meta-replication, i.e., repeating studies across phylogeny, space, and time [27] provides a fundamental advantage over any single-site study, yet has rarely been employed in brood parasitism studies [7, 10, 28, 29]. Additionally we address some poorly studied topics, namely temporal trends in parasitism and their potential causes, and by reviewing all previously collected data on redstart responses to foreign eggs, we assess geographical patterns of redstart defences and their potential causes for the first time. We test the hypothesis that parasitism pressure should positively covary with host defences, namely egg rejection rates [4, 11, 15]. Our work thus enables the common redstart to be employed as a useful model for the study of cuckoo-host co-evolution in the unique cavity-nesting ecological setting.

## Methods

### General procedures

We studied the cuckoo-redstart population nearby Ruokolahti (61°24'N, 28°37'E) in south-eastern Finland during 11 breeding seasons (2006–2016). This study site is ca. 400 km south from the Oulu site studied by Thomson et al. [24] and ca. 160 km south from Joensuu sites studied by Rutila et al. [16]. Additionally, we conducted egg experiments in a non-parasitised redstart population in Bzenec (48°56'N, 17°15'E) in the Czech Republic during 2016. We intentionally created the Bzenec study site as a geographical meta-replicate of our Finnish site (i.e., using identical nest box design and placement in the same habitat, see below). We used the Czech Republic site only for analyses of geographical variation of redstart responses to foreign eggs (see below).

**Fig. 1:**
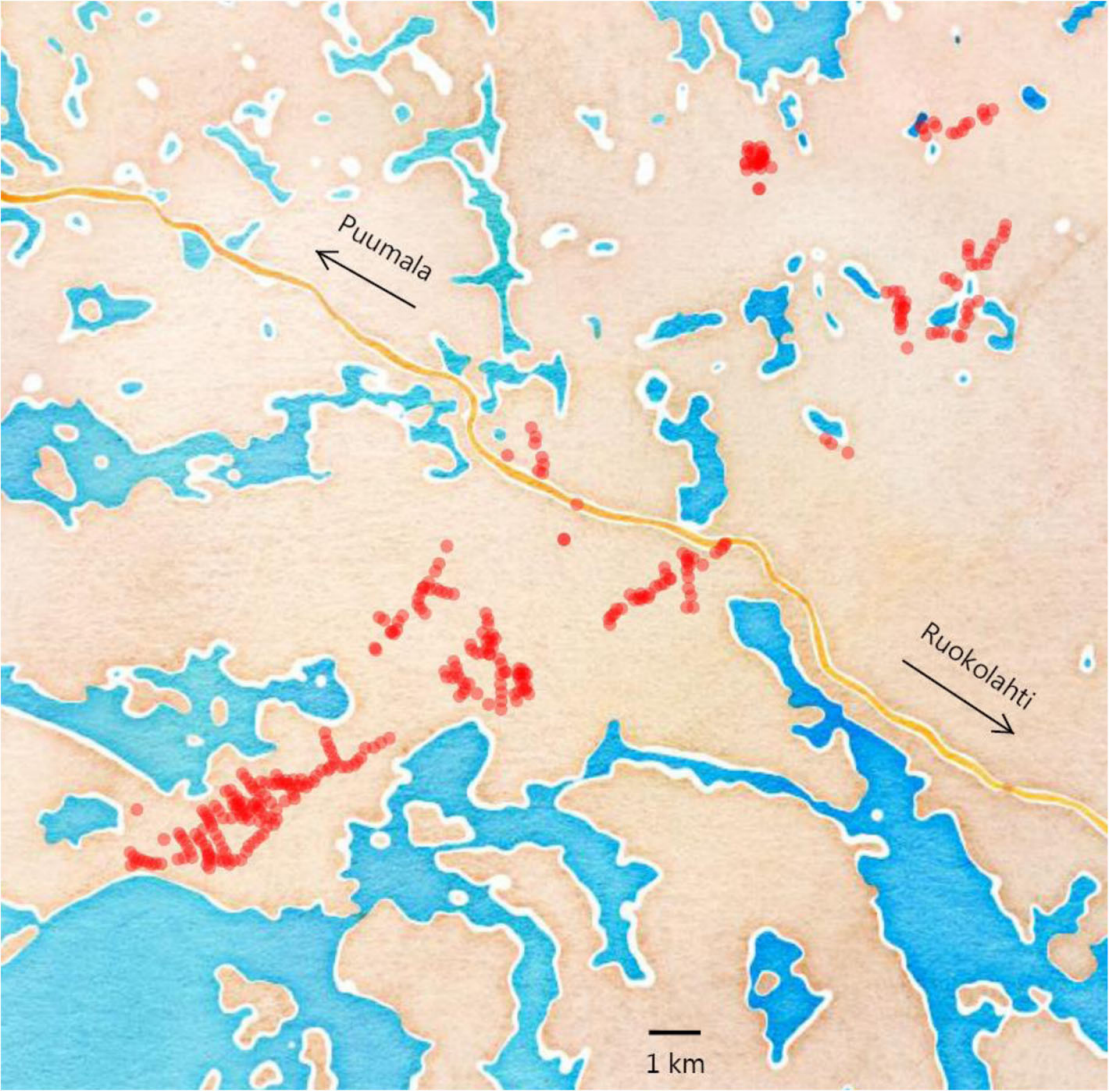
Spatial distribution of nest boxes in Ruokolahti study area spread over 25 x 7 km

Our Finnish study area consisted of multiple sub-sites isolated to varying levels from other sub-sites (Fig. 1). This is important because this micro-geographical spatial meta-replication [27] provides more representative sampling than would be possible with a single site (which is typical for the great majority of ecological studies, including studies of brood parasitism [4]). It is also extremely hard to mist-net cuckoo females because of their secretive behaviour and unwillingness to respond to playback (own unpubl. data); therefore, our spatial design decreases any risks of repeated sampling of eggs and chicks from the same female (pseudoreplication [30]). We note that almost all brood parasitism studies work with non-ringed host populations; and no study, at least in the common cuckoo, has ever directly controlled for parasite female identity [4]. The isolation by space in our study site increases the number of cuckoo females that we sample: an average cuckoo female home range is ~60 ha [9] while our primary study area covers ~12 000 ha (Fig. 1). In contrast, our other study sites [7, 14, 28] are 10–20 times smaller than this. Also the most classic study site among all the study sites where cuckoo research has been done, i.e., Wicken Fen (UK), is more than 20-times smaller than our site [4]. Thus, our study is much more robust against repeated sampling of the same females and their eggs and nestlings than any previous study exactly because of the very unusual spatial scale of our study site.

**Fig. 2:**
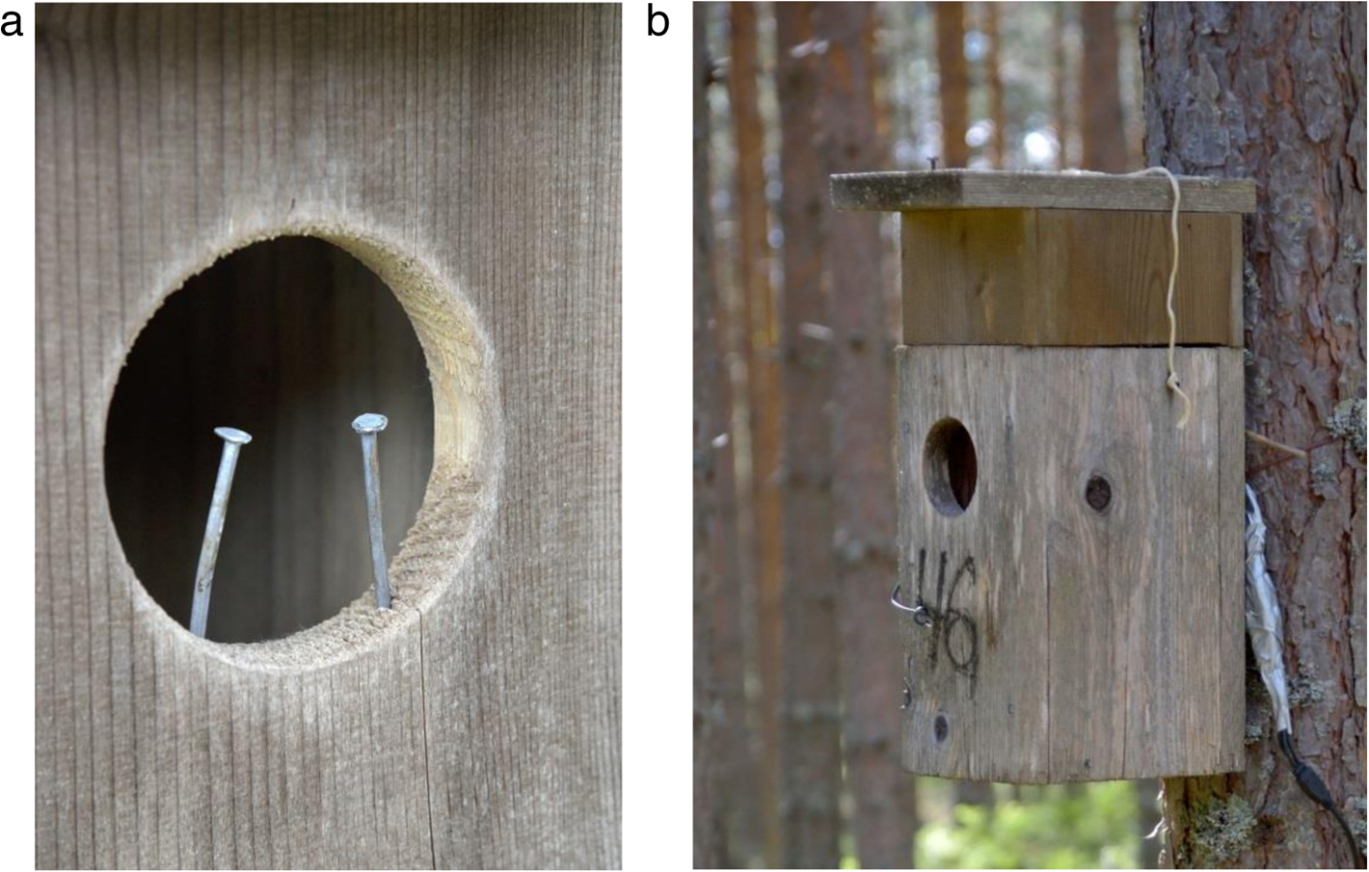
**a** Nails inserted into the nest entrance prevent predator access to the nests. **b** A video-recording box extension. See Methods for explanations. Photo credits: Tomáš Grim

The study sites were covered by forests dominated by cultivated pines (*Pinus sylvestris*). We used ca. 350 nest boxes in Finland and 100 nest boxes in the Czech Republic. All nest boxes were specifically designed for the study of cuckoo-redstart interactions. Nest box inner dimensions were 10–16 x 9–13 cm x 25–32 cm (depth, width and height) with the entrance hole 6–8 cm wide, placed about 1.4–1.7 m above the ground and attached to the tree trunk by wire. Nest boxes were between 50 and 350 m apart, with average distance ca. 100 m. To maximise the data collection we used nails inserted into the nest entrance to prevent predation (Fig. 2a). Nails were inserted after the laying period had finished; nails allowed free movement of redstart parents but prevented predator access. Within parasitised nests, we removed the nails shortly before the estimated fledging time of cuckoos. We cleaned every box after each nesting attempt had finished.

We checked empty nest boxes weekly or bi-weekly. Boxes with active redstart nests were checked several times per week (with the exception of 2011: therefore we did not include 2011 data into some analyses to avoid biases, see below). Around hatching time and when cuckoo nestlings were present in the nest, the boxes were visited daily or every second day. During each nest visit we recorded the presence of parents, their activity (calling, mobbing) and nest content.

Using motion-activated bird box SpyCameraCCTV cameras or Panasonic HDC-HS80 camcorders we video-recorded some nests during the redstart egg-laying stage to determine whether cuckoo eggs found outside the nest cup (see also [16, 24]) were ejected by hosts or mislaid by cuckoos. To minimise disturbance at video-recorded nests we used a video-recording wooden box extension (Fig. 2b; note that at video-recorded nests, we introduced nails only after the host clutch completion, a time when cuckoos rarely lay, see Results).

Local weather data were accessed from open data administered by the Finnish Meteorological Institute [31]. All temperature and rainfall data were obtained from the nearest available weather stations which were located <35 km from our study site.

### Egg experiments

We parasitised redstart nests in our study population by adding one of three types of artificial egg models or five types of real eggs. We manufactured egg models from plaster-of-Paris and painted them with acrylic colours [32]. These artificial egg models weighed between 2.6 and 3.4 g, measured 21 × 16 mm and their size, mass and shape matched those of both the common cuckoo [33] or brown-headed cowbird (*Molothrus ater*) [32]. The blue egg model was painted with a blue colour designed to resemble the cuckoo race which parasitises the redstart [28]. The spotted egg model was creamy-white with brown speckles concentrated around the egg blunt pole, thus resembling a real cowbird egg. The immaculate egg model was creamy-white without spots [32]. Real eggs were represented by real cuckoo eggs (redstart cuckoo race), non-painted conspecific redstart eggs, conspecific redstart eggs painted completely black or painted with black spots [34] and great tit (*Parus major*) eggs painted completely dark blue (Bzenec locality) [35]. We painted eggs with two types of non-toxic permanent markers: Sharpie® for black-coloured eggs and Centropen® for the dark blue colour. Redstart and great tit eggs were collected from freshly abandoned clutches, carefully checked for any cracks, stored in the fridge and used for experiments within 3 days. The term ‘non-cuckoo experimental treatments’ in this study refers to experiments with cowbird, blue, spotted and immaculate models, black-spotted real redstart eggs, complete black redstart eggs and complete dark blue real eggs [32, 34].

Across treatments, we experimentally parasitised the focal nest during the laying (from two eggs laid) or early incubation period (up to day six of incubation) in this study. In two other studies from the same locality [32, 34] nests were parasitised during laying or up to the middle of the incubation stage. Following previous studies (e.g., [6, 7, 24]), experimental eggs were scored as ‘accepted’ if they were present in an active nest (i.e., incubated) at least six days since the egg was introduced and as ‘ejected’ in cases where the experimental egg disappeared while the host own clutch remained incubated. We used the same experimental procedures at both the Finnish and Czech study sites.

Some nests were deserted, but desertion often does not represent a specific response to parasitism because it can result from other unrelated causes (inclement weather, disturbance, etc. [28]). Therefore we also followed randomly-selected control nests which were not used in the experiments with control and experimental nests interspersed in space and time [30]. We followed similar procedures (egg handling, measurements) during nest checks of both experimental and control nests. Control nests included only those nests found during laying or early incubation periods (until 3rd day of incubation), corresponding to the period when egg experiments were conducted and also the period when natural parasitism takes place (see Results). This ensured similar exposure and thus comparable periods between control and parasitised nests.

Hanley et al. [36] found that some hosts (but not others) may elevate their egg rejection responses if they observed the researcher that introduced the foreign egg. Therefore, we included host presence as an additional predictor in our statistical models where we collated this type of information (data from [32] on blue, spotted and immaculate models and data collected in the Czech Republic during 2016). For each experiment we recorded whether the female was present or not when an experimental egg model was introduced by us. We used generalized linear models (binomial distribution, logit link) to examine if female presence (yes or no) predicted host responses. Four egg model types (see above) were included as a categorical predictor. We calculated one model with ejection as a response (i.e., desertion excluded) and second with rejection (i.e., desertion and ejection pooled) as a response.

Further, we extensively checked published studies for reported results on egg discrimination experiments. For each study based on artificial egg models or painted eggs we also retrieved the data on egg type scored subjectively as ‘mimetic’ and ‘non-mimetic’. Although we are aware of the limitations of this approach [37] we could not use a more objective quantification (e.g., via spectrometry) simply because no previous study has provided such data (note that most of the studies were published long before spectrometry started to be used in biological field studies).

### Statistical analyses

**Fig. 3:**
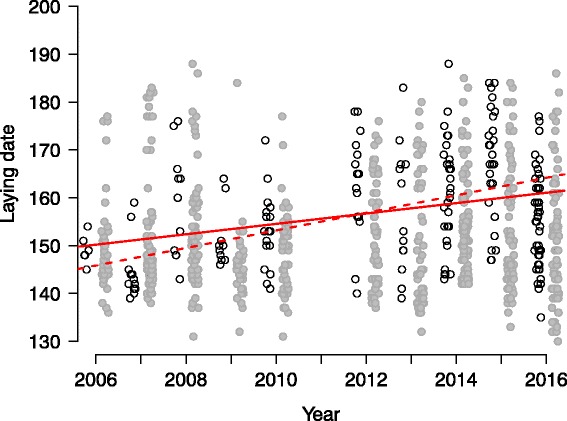
Date of laying (on y-axis 160 = 8th June) by cuckoos (white circles) and redstarts (grey circles). Simple regression lines of date of laying in relation to year for cuckoos (solid line) and redstarts (dashed line; 75th percentile data only, see Methods). Dates of parasitism for season 2011 are not included because of the low frequency of nest checks

We tested for potential temporal trends in cuckoo and redstart egg laying. First, we conducted exploratory analyses of temporal trends in redstart laying from 2006–2016 because cuckoos are limited in their egg laying by available host nests. Potential parameters of host egg laying that could affect the timing of cuckoo egg laying included: median, mean, lower quartile, upper quartile, minimum and maximum of redstart’s first egg-laying dates. We revealed that only the upper quartile (75th percentile) of redstart’s first egg layings (hereafter: redstart FEG upper quartile) showed a significant trend during 2006–2016. Specifically, redstart nests progressed later in the breeding season with the ongoing years (see Fig. 3) while any of other above parameters did not change statistically significantly across years. This result suggests increasing availability of host nests for cuckoos in later breeding seasons within the studied decade. Next, we tested whether the positive trend (see Results) in cuckoo laying could be explained by this increased host nest availability and also by other potential effects of rain and temperature (both continuous; recorded at the day of the parasitism event), or yearly parasitism rate (continuous). We detected high collinearity between the predictors of ‘year’ and ‘redstart FEG upper quartile’ (r = 0.90, variance inflation factor ~9) and therefore included the predictor ‘year’ as a random effect (conservatively modelled as categorical random effect) to control for potential temporal between-year variation.

Further, we tested for potential temporal trends in the hatching success of cuckoo eggs with predictors of ‘year’ (categorical), ‘first egg-laying date of the redstart host’ (continuous), ‘daily rainfall’ (continuous; the mean of daily rainfall values from the start of incubation until hatching) and ‘daily temperature’ (continuous; the mean of average daily air temperature values from the start of incubation until hatching). Similarly, we tested for temporal trends in fledging success of cuckoos using the same predictors as above (rainfall and temperature values were averaged for the period from hatching until fledging of each cuckoo chick) with an additional predictor of ‘brood type’ (binary predictor, cuckoo grew solitarily or shared a nest with redstart chicks in ‘mixed’ broods). We used linear models (responses: cuckoo or redstart egg-laying dates) and generalized linear models (binomial with logit link; responses: hatching and fledging success). We present outputs of both full (as recommended by [38]) and minimum adequate models (obtained via sequential backward elimination of non-significant predictors as recommended by [39]). Potential collinearity among the covariates was low, variance inflation factors were <3 in all cases [40]. We checked the assumption of normality of residual errors, linearity of effect and homogeneity of variances by visual inspection [39].

Sample sizes differed between analyses because some nests were not followed in detail for logistical reasons. Further, deserted and depredated nests could be included for analyses of cuckoo egg fates but needed to be excluded from analyses at nestling stage, for example. Further, sample sizes were lower for cuckoo egg-laying dates than total recorded number of cuckoo layings because we were not able to reliably estimate some laying dates (e.g., intervals were too long between nest checks). Similarly, the precise length of the incubation period (which we compared between cuckoos and redstarts) was not known for a few cuckoo eggs.

For comparisons of parasitism, ejection and desertion rates within this study or between studies (this study versus [16] and [24], respectively), we used Fisher's Exact Tests if sample sizes in any of the categories were close to or below five, otherwise Pearson’s chi-square tests (*χ* ^2^) were used. When calculating Fisher's Exact Test, we added information about an effect size represented by Cohen’s d (standardized mean difference) and its 95% confidence intervals.

We intentionally present some data in an identical way to [24] (same table captions, variable names, data partitioning etc.) to facilitate comparisons. All analyses were conducted in R 3.1.3 (R Core Team 2014) [41].

## Results

### General parasitism characteristics

Across 11 breeding seasons, 32.5% of redstart nests were parasitised by at least one cuckoo egg (Table 1). Overall, we followed 611 active redstart nests but excluded 42 nests that were depredated during the egg-laying period because predation might have pre-empted any chances for late parasitism (Table 1). Parasitism rates varied between 17–50% across years (Table 1). Parasitism rates did not correlate significantly with research effort which was quantified as the number of nest boxes checked per breeding season (Pearson’s r = −0.05, 95% CI = −0.63 to 0.57, *p* = 0.88). In total 17 redstart nests were parasitised multiple times (twice in all cases).

**Table 1:** Number of redstart nests followed in each year and the number parasitised annually

Year	Nests followed	Predated^a^	Nests used	Parasitised (%)
2006	38	7	31	10 (32.3)
2007	37	3	34	13 (38.2)
2008	42	7	35	11 (31.4)
2009	25	4	21	7 (33.3)
2010	34	4	30	15 (50.0)
2011	27	1	26	7 (25.9)
2012	58	1	57	15 (26.3)
2013	75	4	71	12 (16.9)
2014	100	4	96	27 (27.6)
2015	84	2	82	27 (32.9)
2016	91	5	86	41 (47.7)
		Total	569	185 (32.5)
		Effectively parasitised^b^		73 (12.8)

^a^Nests predated during egg laying not allowing us to determine the parasitism status

^b^Parasitised nests where the cuckoo egg was laid in the nest cup

Our study recorded a total of 213 cuckoo eggs laid in our Finnish redstart population. From these, only every third egg was laid into the nest cup (Table 2). The remaining cuckoo eggs were found on the nest rim (i.e., nest material within the nest box but outside the nest cup), on the ground outside the nest box or were even dumped on incomplete nests (Table 2). We did not always check for cuckoo eggs on the ground below redstart nests for logistic reasons (it is often time-consuming to find such mislaid eggs in the dense herbaceous layer around boxes), and thus the number of these eggs could be underestimated. Predators may also remove eggs on the ground prior to our nest box visits. However, even a thorough search for ‘ground’ eggs might not provide reliable estimates because of the complexity of herb vegetation cover and presence of ground cavities. Taking into account multiple parasitism events in some nests, only 12.8% of redstart nests were successfully parasitised from the female cuckoo’s point of view (Table 1).

**Table 2:** The laying site of each cuckoo egg documented in this study

Site of cuckoo egg	Number	Percent
Inside the nest box		
In the nest cup	76	35.7
On the nest rim	116	54.5
Dumped on incomplete nest	11	5.2
On the ground outside	10	4.7
Total	213	

We detected 11 cases where a cuckoo egg was dumped on an incomplete redstart nest containing no eggs. These 11 nests were deserted by redstarts before nest completion and were not included into the overall parasitism rates, or analyses of egg experiments (following the approach of [24]) but were included in an analysis of cuckoo laying dates. We documented six cases when the cuckoo laid her egg after clutch incubation was initiated (one day after host clutch completion in four cases and about middle of incubation period in two cases). The great majority of cuckoo eggs were thus laid during the egg-laying period.

Laying dates of cuckoos were significantly affected by redstart nest availability (Additional file 1). Specifically, when more redstart nests were available later in the breeding season, cuckoos on average parasitised later too (Fig. 3). The cuckoo laying trend was not affected by parasitism rate in a given year and temperature or rainfall at the date of parasitism (Additional file 1).

### Laying stage

We managed to video-record 27 cases of cuckoo-laying at 85 video-recorded nest boxes. Out of 27 eggs, 12 ended up on the nest rim (i.e., in the nest box but outside the nest cup). All these eggs were ignored by redstarts (Table 3). Out of 14 eggs laid into the nest cup, the host redstarts never attempted to grasp or puncture-eject them during the following days when we continued video-recording (mean: 4.7 days, range: 1–7 days).

**Table 3:** The responses of redstarts to artificial egg parasitism experiments and natural parasitism events

Treatment	Number	Ejected	Deserted	Accepted (%)
Manipulated nests				
Control (touch)	89	0	5	84 (94.4)
Mimetic				
Blue model	12	2	2	8 (66.7)
Conspecific	14	0	0	14 (100.0)
Non-mimetic				
Spotted model	13	6	1	6 (46.2)
Immaculate model	7	2	0	5 (71.4)
Spotted own egg	17	0	1	16 (94.1)
Black own egg	22	10	2	10 (45.5)
Blue great tit egg	25	19	0	6 (24.0)
Cuckoo egg	73	0	1	72 (98.6)
Rim cuckoo egg put-in	21	0	2	19 (90.5)
Non-manipulated nests				
Parasitised^a^	43	0	8	35 (81.4)
Non-parasitised^b^	–	–	–	–

All experimental nests are detailed with the number of ejected, deserted and accepted outcomes. We do not have any ‘Excluded’ nests (cf. [24]) because we effectively prevented predation by using nails (see Methods and Fig. 1a). Additional to the mimetic blue ‘redstart’ type model and the non-mimetic spotted (speckled) model (see [24]) we used several other treatments. Data on conspecific eggs (natural host eggs) are from the present study; data on blue, spotted and immaculate (creamy white) models are from [32]; data on own eggs painted with spots or completely black are from [34], and here we additionally included the deserted nests missing in the original study. We use the terms ‘mimetic’ and ‘non-mimetic’ as terms describing the relative similarity between experimental and the host’s own eggs (i.e., not in the absolute objective sense: [37]) and to facilitate the comparison with the same categories as understood by [24]

^a^Effectively parasitised nests where at least one cuckoo egg was naturally laid into the host nest cup

^b^According to our standard protocol that we use in all our studies (e.g., [7, 16, 28, 32, 34]), eggs in all nests were touched, handled and measured, therefore we do not have nests without any manipulation as [24] did

### Incubation stage

We were able to follow the fate of 120 cuckoo eggs (Table 4). In total, 101 (84.2%) were incubated to completion, of which 20 eggs (19.8%) did not hatch (abandoned after parasitism, failure of embryonic development). The hatching success of cuckoo eggs (81 out of 101) was not affected by average rainfall or daily temperatures during the incubation period (Additional file 2: Table S1). In nests with the incubation period length precisely known (*n* = 51; cases with estimated periods excluded), cuckoo eggs hatched earlier (52.9%; mean = 1.3 days, range 1–3 days), on the same day (33.3%) or later than redstart host eggs (13.7%; mean = 1.3, range 1–2).

**Table 4:** The fate of cuckoo eggs followed in relation to different origins

	Total	Eggs incubated to completion	Hatched (unhatched)	Fledged
Naturally laid into nest cup	35	26	22(4)	19
Moved to new nest	68	60	46(14)	39
Rim eggs put into cup	17	15	13(2)	10
Total	120	101	81(20)	68

The duration of the incubation period of cuckoo eggs (mean ± SD, 13.2 ± 1.2, *n* = 51) was significantly shorter than that of redstart eggs in parasitised clutches (13.7 ± 1.2; paired *t*-test: t_50_ = 3.47, *p* = 0.001) but was similar to the incubation period in non-parasitised clutches (13.5 ± 1.0, *n* = 68; Welch’s *t*-test: t_110.6_ = 1.64, *p* = 0.10).

### Nestling stage

Of the 81 cuckoo chicks that hatched, 13 (16.0%) died during the nestling stage. We did not include predation among nestling-fate categories because we used nails (Fig. 2a) to decrease predation rates (predation happened at only seven nests; to avoid biased estimates of predation rates we excluded these cases). Thus, nestling failures in our sample happened due to other reasons than predation (e.g., inclement weather, abandonment by hosts). Consequently, only 67.3% of the cuckoo eggs incubated to completion produced a fledgling (Table 4). Of the original 35 cuckoo eggs successfully laid into the nest cup, only 62.9% hatched and only 54.3% produced a fledgling (Table 4).

Cuckoo chicks evict host eggs or chicks by pushing them onto the rim of the nest [16, 21]. However, not all cuckoo chicks evicted all host young. In 16 of 81 (19.8%) nests, the cuckoo chick was unable to evict all host young and shared the nest with them. These 16 nests with mixed broods (cuckoo and redstart chicks cohabiting) fledged on average 2.4 redstart chicks (range 1–4). In comparison, non-parasitised (and non-predated) redstart nests fledged on average 5.7 redstart chicks (range 0–8). Twelve out of 16 cuckoo chicks that shared the nest cup with redstart nestlings fledged and 55 out of 65 cuckoo chicks that did not share the nest with redstart chicks fledged (Fisher’s Exact Test, *p* = 0.46, Cohen’s d = 0.33 [95% CI = −0.40 to 1.07]). Fledging success also did not differ significantly between solitary cuckoos and cuckoos from mixed broods when controlling for other potential predictors of rainfall, daily temperatures and year (Additional file 1).

### Responses to parasitism: own data

No natural cuckoo egg was ejected by redstart parents (*n* = 137, including eggs cross-fostered by us). The desertion rate of naturally parasitised nests (18.6%) was significantly higher than in control non-parasitised redstart nests followed over the same standard exposure period of six days (5.6%; Fisher’s Exact Test, *p* = 0.03, Cohen’s d = −0.74 [95% CI = −1.40 to −0.09]; Table 3). Similarly, the desertion rate of naturally-parasitised nests was significantly higher than in artificially-parasitised nests by cross-fostering of a real cuckoo egg (1.4%; Fisher’s Exact Test, *p* = 0.001, Cohen’s d = 1.54 [95% CI = 0.36 to 2.72]; Table 3). In contrast, the desertion rate was similar between control nests and those artificially parasitised with a real cuckoo egg by us (Fisher’s Exact Test, *p* = 0.22, Cohen’s d = 0.80 [95% CI = −0.40 to 2.01]; Table 3).

There was large variation in egg ejection rates of non-cuckoo experimental eggs (Table 4). Desertion rates remained rather low in all experimental treatments with non-cuckoo eggs and did not differ among them (Fisher’s Exact Test, *p* = 0.33, Cohen’s d cannot be reliably estimated due to that some treatments show proportions equal to 0). There was also no statistical difference in desertion rates between control versus any of the non-cuckoo experimental treatments (Fisher’s Exact Tests, all *p* > 0.16). We further examined if desertion is a specific response to parasitism in our and [24] populations. First, in our study population, we tested (Fisher’s Exact Tests) desertion rates of control vs mimetic blue model (*p* = 0.16, Cohen’s d = −0.81 [95% CI = −1.88 to 0.26]) and control vs non-mimetic spotted model (*p* = 0.51, Cohen’s d = −0.33 [95% CI = −1.64 to 0.99]) treatments. Similarly, after re-calculating data from [24], there was no difference between control vs mimetic (*p* = 1.00, Cohen’s d cannot be reliably estimated due to one of the treatments shows proportion equal to 0) and non-mimetic (*p* = 0.62, Cohen’s d = −0.62 [95% CI = −1.91 to 0.67]) treatments in their Oulu population. Desertion is therefore not a response to experimental parasitism (in both our and [24] study sites). Ejection rates (i.e., desertions excluded) in our vs [24] study populations did not differ for the mimetic experimental treatment (Fisher’s Exact Test, *p* = 0.14, Cohen’s d cannot be reliably estimated due to one of the treatments shows proportion equal to 0) but differed for the non-mimetic one (*p* = 0.001, Cohen’s d = −1.59 [95% CI = −2.62 to −0.56]; Tables 3 and 5).

**Table 5:** Review of redstart responses to foreign eggs

Model	Locality	Number	Ejected (%)	Deserted (%)	Parasitism rate	Reference
Natural cuckoo eggs						
Non-manipulated	Finland NW	46	0	13	31	[24]
Non-manipulated	Finland E	54	0	13	21	[16]
Non-manipulated	Finland C	187	0	4	44	[48]
Non-manipulated	Czech Republic	62	0	6	31	[20]
Non-manipulated	Finland SE	43	0	19	33	this study
Cross-fostered	Finland NW	16	0	0	31	[24]
Cross-fostered	Finland SE	73	0	1	30	this study^a^
Conspecific eggs	Finland SE	14	0	0	30	this study
Mimetic models						
Redstart-cuckoo type	Finland NW	16	0	0	31	[24]
Redstart-cuckoo type	Finland NC	9	11	33	0	[25]
Redstart-cuckoo type	Finland E	26	4	4	21	[16]
Redstart-cuckoo type	Finland E	29	3	3	17	[25]
Redstart-cuckoo type	Great Britain	1	0	0	0	[5]
Cowbird blue	Finland SE	12	17	17	30	[32]
Non-mimetic models						
Brambling-cuckoo	Finland NW	41	5	7	31	[24]
Brambling-cuckoo	Finland NC	14	14	36	0	[25]
Brambling-cuckoo	Finland E	27	41	4	21	[16]
Brambling-cuckoo	Finland E	37	32	5	17	[25]
Meadow pipit-cuckoo	Norway C	5	0	20	0	[6]
Meadow pipit-cuckoo	Great Britain	4	0	0	0	[5]
Pied wagtail-cuckoo	Great Britain	4	50	0	0	[5]
Cowbird spotted	Finland SE	13	46	8	30	[32]
Cowbird immaculate	Finland SE	7	29	0	30	[32]
Non-mimetic natural eggs						
Black complete	Finland SE	22	45	9	30	[34]
Black spots	Finland SE	17	0	6	30	[34]
Blue complete	Czech Republic	25	76	0	0	this study^b^
Great tit	Finland SW	7	57	0	0	[50]
Various species	Finland SW	6	0	0	0	[42]^c^
Various species	Finland N	35	31	0	0	[43]^d^

See original studies for detailed information on the size, colour and maculation of experimental eggs

^a^Sample sizes differ from Table 3 because desertion was not a specific response to parasitism in this treatment (Table 3, Results); therefore we excluded the deserted nests

^b^See Methods

^c^Author used natural eggs of the chaffinch, redwing, great tit and wryneck; experimental eggs introduced around hatching time

^d^Author used natural eggs of the brambling, meadow pipit and redpoll; acceptance period was set to 24 h, then the experimental egg was removed

Presence of the redstart female during experimental parasitism did not significantly influence responses to experimental eggs when desertions were included (*χ* ^2^ = 0.66, *p* = 0.42) or excluded (only ejection as a host's response: *χ* ^2^ = 0.51, *p* = 0.47).

### Responses to parasitism: literature review

Rejection rates were significantly higher in non-manipulated natural cuckoo eggs compared to cross-fostered ones (*χ* ^2^ = 9.8, *p* = 0.002) and the probability of rejection increased with increasing parasitism rate (*χ* ^2^ = 8.0, *p* = 0.005; Table 5). Both rejection (*χ* ^2^ = 40.9, *p* < 0.001) and ejection (*χ* ^2^ = 109.8, *p* < 0.001) rates differed between five treatments when controlling for the significant effect of parasitism rate (Table 5). Specifically, increasing natural parasitism rates led to an increase in rejection rates (i.e., ejection and desertion pooled: *χ* ^2^ = 9.09, *p* = 0.003) and marginally non-significantly in ejection rates (*χ* ^2^ = 2.9, *p* = 0.09; Table 5). Excluding data from the methodologically different studies [42, 43] did not change the conclusions, except that ejection rates significantly increased with natural parasitism rates (*χ* ^2^ = 8.1, *p* = 0.004).

## Discussion

We found high rates of cuckoo parasitism in our study population with about every third nest parasitised naturally. Almost all cuckoo eggs were laid during the host egg-laying stage. However, only every third cuckoo egg was laid into the host nest cup. Some eggs even ended up outside boxes on the ground. Thus, cavity nesting seems to be fatal for two thirds of cuckoo laying attempts, lowering the parasite's breeding success radically. We acknowledge that our data are from artificial nest boxes (just like in the vast majority of published studies of any cavity nesting passerines; for discussion, see [35, 44]). Despite substantial attempts we could not find any natural cavities that could be video-recorded at laying and incubation stage – all natural cavities we found were detected due to loud begging calls of chicks, thus making any conclusions about egg laying impossible (own observations, J. Haikola, M. Kysučan, pers. comm.). However, the inner sizes of nest boxes used in this study were similar to inner sizes of natural cavities [23]. Therefore we believe our conclusions based on boxes roughly reflect the biological reality of natural cavities.

Our results are quantitatively remarkably similar to those recently reported from detailed research at another study site in Finland [24]. The parasitism rate was virtually identical in Ruokolahti (32.5%) and Oulu (31.1%). For comparison, parasitism rates in the most frequently studied open nesting species, reed warblers (*Acrocephalus scirpaceus*), varied from 0.0 to 21.1% (data from 16 European populations [10]). Also the laying sites of cuckoo eggs (Table 2) were proportionally virtually the same as those found in Oulu (Table 2 in [24], Table 6). However, these results differed from the sites studied by [16] where the parasitism rate was 20.6% and more eggs ended up in the nest cup (52.9%) than on the nest rim (33.3%; Table 6).

**Table 6:** Statistical comparison of results of this study with previous intensive studies of redstart-cuckoos

Parameter (%)	Rutila et al. (2002) [16]		Thomson et al. (2016) [24]	
	*χ* ^2^ (df = 1)	*p*	*χ* ^2^ (df = 1)	*p*
Overall parasitism rate	16.32	<0.001	0.20	0.66
Effectively parasitised nests	0.30	0.58	0.001	0.98
Cuckoo egg in the nest cup	8.48	0.004	0.26	0.61
Cuckoo egg on the nest rim	12.34	<0.001	0.03	0.86
Cuckoo egg dumped on incomplete nest	0.87	0.35	0.79	0.38
Cuckoo egg on the ground outside	0.20	0.65	0.08	0.77
Mimetic egg rejection	–	0.19	–	0.14
Non-mimetic egg rejection	–	0.73	–	0.001

Data for parasitism rate and egg positions were analysed with Pearson’s chi-square test, host rejection rates (mimetic egg = blue model, non-mimetic egg = spotted model) were analysed with Fisher’s Exact Test (all df = 1). Desertion was not a specific response to foreign eggs (Results) and was therefore excluded

We detected a clear temporal trend in cuckoo egg laying: with advancing years, cuckoos laid significantly later. This trend was explained by host laying dates: because redstarts extended their laying dates across years into the late summer they extended the period when their nests were available to cuckoos. This led to increased average egg-laying dates in cuckoos (but not in redstarts). This trend was explained neither by weather conditions (cf. [45]) nor yearly parasitism rates.

### Host egg rejection

All nests that were parasitised too early (cuckoos laid before any host eggs appeared in the nest), were deserted by hosts. Cuckoo eggs that were laid synchronously (during the host laying period) were never ejected by hosts and we did not find any signs of unsuccessful puncture attempts (cf. [13]). Still, desertion rates at parasitised nests were much higher than background desertion rates at non-parasitised nests; also cuckoo eggs cross-fostered by us were deserted rarely and similarly to non-parasitised nests. These patterns support the hypothesis that the cue triggering egg discrimination in redstarts is primarily the sight of the adult cuckoo at the nest in both redstarts [24] and some other cuckoo hosts [46].

Why do redstarts not eject natural parasite eggs? Cuckoo eggs show perfect mimicry [17] thus pre-empting egg ejection as a viable host defence strategy [24]. This may explain why redstarts only desert some parasitised nests, most likely those where they witnessed the cuckoo female laying. Discrimination of the cuckoo female as a specific threat is also indirectly supported by observation of higher foreign-egg rejection rates in redstart populations that have higher parasitism rates (see Results). Further, we found that while redstarts deserted naturally-parasitised nests, they did not desert nests with cuckoo eggs introduced by an experimenter. Interestingly, witnessing an experimenter during experimental parasitism by the redstart female did not influence her response which supports the pattern that observing an experimenter by a host female affects host responses only in some host species [36].

Similar to parasitism rates (above), host egg rejection rates also show remarkably low variation across redstart populations (considered for each experimental egg treatment separately, to avoid confusing the effect of host-parasite egg similarity). Naturally-laid cuckoo eggs were never ejected by redstarts in any population studied so far (Table 6). There was little variation (from 13% to 18%) in rejection rates (by desertion) within the same populations. In contrast, egg rejection rates (by both ejection and desertion in most cases) of non-mimetic experimental eggs intraspecifically varied three- to five-fold in several other hosts [7, 11, 28]. Such variation in egg rejection rates is rather a rule than an exception. For example, a review of several actual or potential cuckoo hosts demonstrates two- to five-fold difference in rejection rates within species where experiments were standardized by using the same methodology (Table 1 in [47]).

All previous studies used indirect experimental evidence to support the hypothesis that redstarts do not eject naturally-laid cuckoo eggs and that cuckoo eggs found on the nest rim are not ejected by hosts but mislaid by the parasite. Our study is the first to provide direct (video-recording) evidence that redstarts never ejected any naturally-laid cuckoo eggs. All eggs that appeared on the nest rim in the video-recorded boxes were mislaid by cuckoos. This is in line with extensive – but indirect – experimental evidence and shows that the inference that cuckoo eggs found on the nest rim were ejected by redstarts [48] was flawed.

### Cuckoo success

Eviction success was similar (80% vs. 85%) to that found by [24] whereas [16] reported a much lower success of 54%. But whether this variation reflects population-specific nest cup design [7, 21] remains to be tested. In contrast to a previous experimental study [21] but similar to observational studies [16, 24] we did not find decreased fitness in cuckoo chicks from mixed broods. However, it remains to be explored whether cuckoos from mixed broods suffer delayed costs (e.g. lower fledging mass, higher fledging age, decreased parental post-fledging care) which can cause decreased survival in the post-fledging stage or in later life stages.

Because of cumulative effects of mislaying, hatching failures and cohabitation with hosts chicks the overall cuckoos’ breeding success (number of young fledged per egg laid) was 16%. This value is similar to 18% found by [16], 14% reported by [24], and 11% published by [48]. Still, cuckoo fitness may be much lower in regular cuckoo hosts (e.g. only 4% in the marsh warbler *Acrocephalus palustris*, [8]). Based on this estimate of breeding success and assuming that a cuckoo lays 10–20 eggs per season [49], the fitness of one adult redstart-cuckoo female represents only 1–3 successful cuckoo chicks per season.

## Conclusions

Overall, our results on many aspects of cuckoo-redstart biology are quantitatively very similar to those from two other study sites where detailed and similarly designed studies were done [16, 24]. Thus, our meta-replication approach suggests that results from any of these populations might be reasonably generalised to other sites (for a similar case see [7]). Our study also provides a necessary baseline in natural history data for future more detailed observational and experimental studies of this unusual host-parasite system.

Yet, our data are not from natural cavities but from artificial nest boxes. Our boxes were specifically designed to be similar, as for both entrance sizes and inner dimensions, to natural cavities [23]. We stress that, from logistic reasons, all other cavity nesters (tits, flycatchers, nuthatches, etc.) are also studied only or at least mostly in nest boxes. Our study population has been regularly parasitized and studied for almost four decades [19] which represents one of the longest (if not the longest) continuous records of parasitism in any brood parasite-host system globally; thus, before the start of the present study cuckoos had sufficient time to get used to artificial nest boxes. This is best evidenced by very high parasitism rates in our study population. Still, future studies should also focus, additionally to nest boxes, on natural cavities. Although this will be logistically very challenging, such an endeavour might be also very rewarding.

The hole-nesting ecological context has typically been ignored in both primary studies (see Introduction) and reviews (e.g. [1]). We hope that the present and other recent studies [24] will elicit more research on the potential effects of cavity nesting on other aspects of cuckoo-host co-evolution because both abiotic (cavity microclimate) and biotic parameters (cohabitation of cuckoo and host chicks) are not paralleled in any other cuckoo host and thus provide an ideal natural test of flexibility and limits of cuckoo biology.

## Additional files

Additional file 1: Output of models explaining variation in cuckoo laying date, hatching success and fledging success. (DOCX 24 kb)
Additional file 2: Raw dataset used in this study. (XLSX 43 kb)
