# Supplementary material for: The common redstart as a suitable model to study cuckoo-host coevolution in a unique ecological context
Source: BMC Evol Biol. 2016 Nov 25;16:255. doi: 10.1186/s12862-016-0835-5 (PMC5124271; doi:10.1186/s12862-016-0835-5)
Supplement: Additional file 1: — Output of models explaining variation in cuckoo laying date, hatching success and fledging success. (DOCX 24 kb) [file 12862_2016_835_MOESM1_ESM.docx]

**Additional file 1**

**The common redstart as a suitable model to study cuckoo-host coevolution in a unique ecological context**

Peter Samaš, Jarkko Rutila and Tomáš Grim

Outputs of the full models (FM) and the minimum adequate (MAM) models for effects (all continuous) of yearly 75^th^ percentile of redstart FEG (first egg-laying date), yearly parasitism rate, rainfall and daily temperatures on the response variable of cuckoo laying date (continuous) and effects of year (categorical), redstart FEG (continuous), rainfall and temperature (continuous) on the binary response variables of hatching success of cuckoo eggs and fledging success of cuckoo chicks (binomial GLMs). In the analysis of laying date we included year as a random effect to control for potential confounding effect of between-year variation (see also Methods). In the analyses of hatching success and fledging age we had sufficient data only for breeding seasons 2012–2016. The additional predictor of brood type (binary; cuckoo grew solitary or shared the nest with redstarts) was included for predicting fledging success. Rainfall and daily temperatures were averaged over incubation and nestling stages for responses of hatching success and fledging success, respectively.

| Predictor | FM | | |  | MAM | | |
| --- | --- | --- | --- | --- | --- | --- | --- |
|  | χ^2^ | p | Estimate±SE |  | χ^2^ | p | Estimate±SE |
| **Laying date** |  |  |  |  |  |  |  |
| Intercept | – | – | 67.1±41.8 |  | – | – | 55.3±37.3 |
| Redstart FEG | 5.75 | 0.02 | 0.60±0.26 |  | 6.50 | 0.01 | 0.65±0.24 |
| Parasitism rate | 1.05 | 0.30 | −0.16±0.18 |  | – | – | – |
| Rainfall | 3.69 | 0.06 | 0.42±0.21 |  | – | – | – |
| Temperature | 0.04 | 0.84 | 0.07±0.23 |  | – | – | – |
|  |  |  |  |  |  |  |  |
| **Hatching success** |  |  |  |  |  |  |  |
| Intercept | – | – | 5.35±4.11 |  | – | – | 1.36±0.26 |
| Year (df=4) | 4.05 | 0.40 | – |  | – | – | – |
| FEG | 1.57 | 0.21 | −0.04±0.03 |  |  |  |  |
| Rainfall | 0.28 | 0.59 | 0.11±0.21 |  | – | – | – |
| Temperature | 1.53 | 0.22 | 0.19±0.16 |  | – | – | – |
|  |  |  |  |  |  |  |  |
| **Fledging success** |  |  |  |  |  |  |  |
| Intercept | – | – | 48.41±23.66 |  | – | – | 22.60±8.80 |
| Brood type | 1.66 | 0.20 | −2.00±1.55 |  | – | – | – |
| Year (df=4) | 4.71 | 0.32 | – |  | – | – | – |
| FEG | 5.16 | 0.02 | −0.18±0.11 |  | 4.94 | 0.03 | −0.06±0.03 |
| Rainfall | 3.81 | 0.05 | −0.80±0.61 |  | – | – | – |
| Temperature | 0.97 | 0.32 | −0.54±0.66 |  | – | – | – |
